# Successful GH Treatment of Hepatopulmonary Syndrome in Panhypopituitarism-related Advanced Liver Disease

**DOI:** 10.1210/jcemcr/luaf307

**Published:** 2026-03-26

**Authors:** Stephanie Chen, Rodrigo Diaz-Lankenau, Allison Kwong, Julia Chang, James McAvoy, Yu Kuang Lai

**Affiliations:** Division of Pulmonary, Allergy, and Critical Care Medicine, Stanford University, Stanford, CA 94305, USA; Division of Pulmonary, Allergy, and Critical Care Medicine, Stanford University, Stanford, CA 94305, USA; Division of Gastroenterology and Hepatology, Stanford University, Stanford, CA 94305, USA; Division of Endocrinology, Gerontology, & Metabolism, Stanford University, Stanford, CA 94305, USA; Division of Anesthesiology, Perioperative and Pain Medicine, Stanford University, Stanford, CA 94305, USA; Division of Pulmonary, Allergy, and Critical Care Medicine, Stanford University, Stanford, CA 94305, USA

**Keywords:** case report, hepatopulmonary syndrome, panhypopituitarism, growth hormone

## Abstract

Hepatopulmonary syndrome (HPS) is a known pulmonary vascular complication of chronic liver disease. In rare circumstances, HPS has been described in the context of panhypopituitarism. An underlying mechanism of panhypopituitarism-related liver injury is thought to stem from GH deficiency, leading to steatohepatitis from augmented lipid deposition within hepatocytes. Although liver transplantation remains the definitive treatment for HPS, resolution of panhypopituitarism-related HPS following GH replacement therapy has been occasionally described. These successful cases uniformly showed hepatic steatosis on biopsy that resolved after GH replacement, suggesting GH may effectively reverse the pathological process before permanent damage occurs. We present the first reported case of panhypopituitarism-related HPS successfully treated with GH replacement in the presence of significant liver fibrosis without steatosis. This case highlights the sustained therapeutic efficacy of GH even in advanced liver disease and adds to the limited literature regarding successful treatment of HPS, especially in the context of panhypopituitarism, without liver transplantation.

## Introduction

Hepatopulmonary syndrome (HPS) is a rare complication of metabolic dysfunction-associated steatotic liver disease (MASLD), which may develop in some patients with panhypopituitarism and untreated GH deficiency (GHD). Currently, the only established treatment for HPS is liver transplantation (LT). We present a unique case of panhypopituitarism-related HPS in the context of hepatic fibrosis that was successfully treated with GH replacement alone.

## Case Presentation

A 23-year-old male with congenital panhypopituitarism presented with acute on chronic dyspnea and lethargy. On evaluation, he was febrile to 100.3 °F (37.9 °C), bradycardic (60 beats/min), hypotensive (80/40 mm Hg), and hypoxemic with oxygen saturation (SpO_2_) of 43% on room air requiring high-flow nasal cannula at 50 L/min and fraction of inspired oxygen value of 90% to maintain SpO_2_ above 90%. His weight was 182 lb (83 kg) and height was 6′3″ (1.9 m), with a body mass index of 24.82 kg/m^2^. He had been lost to follow-up with endocrinology and off GH replacement for at least 6 years with unclear adherence to glucocorticoid and thyroid hormone replacement. He denied any tobacco, alcohol, or recreational drug use. He had no significant family history.

Examination revealed clubbing, cyanosis, clear lung sounds, and orthodeoxia. Laboratory results confirmed panhypopituitarism, along with elevated aminotransferases and an abnormal arterial blood gas with room air arterial partial pressure of oxygen of 50.6 mm Hg (Table 1). Chest computed tomography scans showed mild multifocal ground-glass opacities without evidence of pulmonary embolism. Infectious workup was positive for influenza A.

**Table 1.** Clinical data at initial hospitalization and 3 months after GH therapy

Parameters	Initial hospitalization	After GH therapy	Reference range
PaO_2_ on room air	50.6 mm Hg (6.7 kPa)	82.9 mm Hg (11.1 kPa)	80-105 mm Hg (10.7-14 kPa)
A-a gradient on room air	48.1 mm Hg (6.4 kPa)	15.8 mm Hg (2.1 kPa)	<9.8 mm Hg (<1.3 kPa)
Free T4	0.14 ng/dL (1.8 pmol/L)	1.38 ng/dL (17.8 pmol/L)	No levothyroxine: 0.9-1.70 ng/dL (12-22 pmol/L) On levothyroxine: 0.9-2.10 ng/dL (12-27 pmol/L)
IGF-1 level	<10 ng/mL (<1.3 nmol/L)	85 ng/mL*^a^* (11.1 nmol/L)	66-346 ng/mL (8.6-45.2 nmol/L)
Albumin	3.6 g/dL (36 g/L)	4.1 g/dL (41 g/L)	3.5-5.2 g/dL (35-52 g/L)
AST	111 U/L (1.8 μkat/L)	85 U/L (1.4 μkat/L)	10-50 U/L (0.2-0.8 μkat/L)
ALT	260 U/L (4.3 μkat/L)	173 U/L (2.9 μkat/L)	10-50 U/L (0.2-0.8 μkat/L)
Total bilirubin	1.1 mg/dL (18.8 μmol/L)	0.6 mg/dL (10.3 μmol/L)	<1.2 mg/dL (<20.5 μmol/L)
Platelet count	92 K/μL (92 G/L)	177 K/μL (177 G/L)	150-400 K/μL (150-400 G/L)
MAA scan	61% shunt fraction	7.6% shunt fraction*^b^*	<7% shunt fraction

Abbreviations: A-a gradient, alveolar arterial oxygen gradient; ALT, alanine aminotransferase; AST, aspartate aminotransferase; MAA scan, macroaggregated albumin scan; PaO_2_, arterial partial pressure of oxygen; TTE, transthoracic echocardiogram.

^*a*^6 weeks after GH therapy.

^*b*^6 months after GH therapy.

## Diagnostic Assessment

His initial laboratory testing was notable for low free T4 (FT4) of 0.14 ng/dL (SI: 1.80 pmol/L) (reference range, 0.93-1.70 ng/dL [SI: 12-22 pmol/L]) and low IGF-1 of less than 10 ng/mL (SI: <1.3 nmol/L) (reference range, 66-346 ng/mL [SI: 8.6-45.2 nmol/L]) (Table 1). Testosterone levels were not drawn as they were presumed to be low from his critical illness, and he was not on testosterone therapy. A previous testosterone level 6 years prior to admission was less than 10 ng/dL (SI: <0.35 nmol/L) (reference range, 250-1000 ng/dL [SI: 8.7-34.7 nmol/L] for adult males).

**Figure 1.**
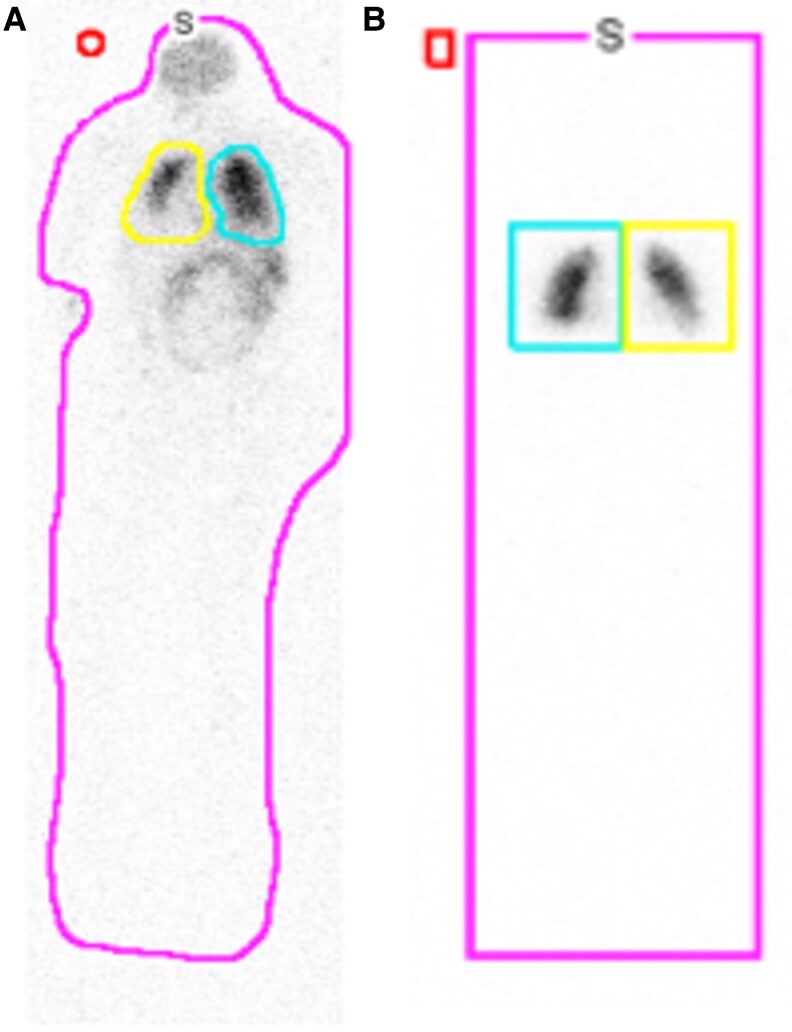
(A) Initial technetium-labeled macroaggregated albumin scan with abnormally increased radiotracer activity in brain, liver, kidneys, and loops of bowel consistent with significant right to left shunt. Shunt fraction was measured to be 61.4% (normal is <7%). (B) Technetium-labeled macroaggregated albumin scan 6 months after GH treatment. No radiotracer activity is seen in brain, liver, or kidneys and right to left shunt is no longer visually present. Shunt fraction was measured to be 7.6%.

Given the severe hypoxemia disproportional to imaging findings, a contrast-enhanced echocardiography was obtained showing a large intrapulmonary shunt, further quantified by technetium-labeled macroaggregated albumin scan at 61% (Fig. 1A). Liver biopsy demonstrated bridging fibrosis with focal nodularity (stage 3-4 of 4) and no steatosis. The underlying etiology for the fibrosis was not obvious, but the focal, patchy pericellular fibrosis was potentially consistent with “burnt-out” steatohepatitis [1]. Following a comprehensive workup excluding other causes of liver disease, the patient was diagnosed with advanced liver fibrosis, presumed secondary to “burnt-out” MASLD from longstanding panhypopituitarism, now complicated by HPS.

## Treatment

The patient was treated with oral oseltamivir 75 mg twice per day for 5 days. He was simultaneously started on IV hydrocortisone 50 mg three times per day, IV levothyroxine 100 mcg daily, and IV liothyronine 5 mcg twice per day given shock state. As his blood pressure improved, the patient was weaned to oral hydrocortisone 20 mg twice per day and thyroid replacement changed to oral levothyroxine 125 mcg daily, with discontinuation of IV liothyronine. FT4 levels improved to normal range within 2 weeks of restarting levothyroxine replacement. He remained on a regular diet throughout hospitalization. Despite resolution of parenchymal opacities and shock, his hypoxemia persisted, prompting a multidisciplinary discussion regarding LT. However, the severity of his hypoxemia posed a high likelihood of requiring extracorporeal membrane oxygenation (ECMO) support. Balancing the potential reversal of HPS with GH replacement reported in the literature against the substantial perioperative risk, a shared decision was made to proceed with a trial of recombinant human GH (rhGH) therapy to minimize perioperative risk by improving hypoxemia. Subcutaneous rhGH was started at 1.0 mg daily about 1 month into admission, consistent with rhGH therapy doses used in previously published case reports for panhypopituitarism-related MASLD [2-4].

## Outcome and Follow-up

Six weeks after initiation of rhGH, his IGF-1 level improved to 85 ng/mL (SI: 11.1 nmol/L) and FT4 levels remained within normal limits. Three months after rhGH therapy initiation, his SpO_2_ improved to 95% on room air and 89% with exertion. He had near-normalized arterial blood gas results, along with improvement in aminotransferases (Table 1). Six months after rhGH therapy initiation, his SpO_2_ improved to 98% on room air and 95% with exertion. Repeat technetium-labeled macroaggregated albumin scan at this time showed improvement in shunt fraction to 7.6% (Fig. 1B). Given significant improvement in hypoxemia and intrapulmonary shunting, LT was deferred for continued close clinical monitoring. He is now on oral hydrocortisone 15 mg in the morning and 10 mg in the afternoon, oral levothyroxine 125 mcg daily, and subcutaneous rhGH replacement 1.3 mg daily with dose adjustments made based IGF-1 and FT4 laboratory values. Although IGF binding-protein-3 has been proposed to have a role in diagnosis of GHD and monitoring of GH replacement therapy in children, its value in adults in unclear, and conditions that may additionally lower IGF-1 (eg, malnutrition, ongoing hypothyroidism, renal failure) were not present in this patient [5, 6]. Thus, only IGF-1 has been used as the marker during diagnosis and monitoring for this patient at this time.

## Discussion

Panhypopituitarism with GHD is associated with central obesity, insulin resistance, and dyslipidemia with subsequent development of MASLD. GH is crucial in regulating lipid deposition within hepatocytes and stimulating the production of IGF-1. Both GH and IGF-1 are also known for their anti-inflammatory and antifibrotic properties. Disruption in GH and IGF-1 signaling pathways have been shown to lead to significant hepatic steatosis in rodent models. Compelling evidence in both rodent and human models have demonstrated their efficacy in reversing hepatic steatosis, improving inflammation, and preventing progression of fibrosis [7]. There have been a couple of proposed mechanisms for GH's therapeutic benefit. GH is thought to increase nutrient utilization, muscle mass, and resting energy expenditure with decrease in visceral adipose tissue, leading to prevention of steatosis. In skeletal muscle, GH appears to mediate supply of nonesterified fatty acids for use over glucose, thus enhancing whole-body fatty acid oxidation because of substrate availability. GH has also been shown to decrease markers of inflammation, thought partly because of its direct action on immune cell function, switching macrophages from an inflammatory profile to an anti-inflammatory profile [8]. Therefore, there is growing research into GH's therapeutic role in MASLD [7, 9].

Glucocorticoids (GC) also play an important role in liver physiology by stimulating gluconeogenesis, promoting glycogenolysis, and regulating cholesterol and fatty acid synthesis [10]. Furthermore, GC suppress excessive immune response and inflammatory reactions, which are often present in liver failure. They are thought to exhibit a stabilizing effect on cell membranes by preventing hepatocyte disintegration and slowing the course of liver damage. Therefore, GC have also been used for treatment of liver failure in certain etiologies, such as alcoholic hepatitis, autoimmune hepatitis, and drug-induced liver injury [11].

HPS is a well-known pulmonary vascular complication characterized by intrapulmonary vascular dilation causing shunt physiology, leading to impaired oxygenation in the setting of liver disease, portal hypertension, or congenital portosystemic shunt [12]. Clinical manifestations include respiratory and systemic symptoms superimposed from liver disease, often manifesting as dyspnea and hypoxemia that severely impair daily activity and quality of life [13, 14]. Although numerous mechanistic pathways have been implicated in the pathophysiology of HPS, trials or case series targeting these specific mechanisms have not consistently demonstrated clinical benefit [12]. Consequently, LT remains the only cure for HPS. The prognosis for patients with HPS without LT is guarded, with a 5-year survival of 23% [15]. Reassuringly, patients with HPS who undergo LT have favorable outcomes comparable to LT recipients without HPS [16].

Post-LT hypoxemia is a feared complication and a leading cause of prolonged hospitalization and post-LT mortality. This risk is particularly pronounced in patients with very severe HPS like our case (defined as having a pretransplant arterial partial pressure of oxygen of less than 50 mm Hg on room air) [17, 18]. The management of post-LT hypoxemia is primarily supportive while waiting for the gradual resolution of pulmonary vasculopathy. In severe instances, refractory hypoxemia may necessitate prolonged mechanical support including ECMO, exposing the patient to ECMO-related complications and further increasing mortality risk [19]. Given the complex and substantial perioperative risks, some centers are reluctant to offer LT and referral to experienced LT centers is recommended.

Limited cases have demonstrated successful reversal of HPS without the need for LT when the etiology of liver insult is addressed (Table 2). GH replacement has shown similar success in treating HPS secondary to panhypopituitarism-related MASLD [20]. Notably, prior cases uniformly had histological confirmation of steatosis with varying degree of inflammation and fibrosis, suggesting the potential for disease reversal. Although the presence of advanced hepatic fibrosis in our case raised the concern for disease irreversibility, we postulate that the anti-inflammatory property of GH reversed residual inflammation driving the patient's fibrosis and led to an improvement in HPS.

**Table 2.** Existing literature regarding resolution of hepatopulmonary syndrome without liver transplantation

Reference	Number of cases	Etiology of HPS	Severity of HPS^a^	Intervention
Regev et al [24]	1	Acute hepatitis A	Severe	Supportive care while awaiting resolution of hepatitis A ledto resolution of HPS
Tzovares et al [25]	1	Granulomatous hepatitis	Severe	Methylprednisolone 32mg daily with complete reversion of HPS
De et al [26]	2	Budd-Chiari syndrome	Moderate	Patient 1 treated with cavoplasty with reversal of HPS; patient 2 with unsuccessful attempt at cavoplasty and no change in HPS
Choe et al [20]	10	Hypopituitarism of different etiologies	Various	7 patients treated with GH replacement. 6 patients reporting improvement and 4 patients no longer requiring liver transplant; 1 patient treated with weight loss and calorie reduction with resolution of HPS

Abbreviations: GH, growth hormone; HPS, hepatopulmonary syndrome; PaO_2_, arterial partial pressure of oxygen.

^a^Mild: PaO_2_ ≥ 80 mmHg; moderate: PaO_2_ 60-79 mmHg; severe: PaO_2_ < 60 mmHg.

Although our patient was also started on GC for treatment of adrenal insufficiency, we do not believe this had a significant effect on his HPS improvement. GC have been used in treatment of certain etiologies of liver failure; however, they have been unsuccessful for treatment of HPS [21]. Furthermore, his oxygen saturation did not improve after 1 month of GC and levothyroxine replacement despite normal free T4 levels, suggesting that GH replacement was the primary driver of his HPS clinical improvement.

During shared decision making for trial of rhGH, concern was raised regarding the increased mortality associated with GH therapy in critically ill adults. rhGH therapy has been associated with increased length of hospitalization (including intensive care stay) and duration of mechanical ventilation. The mechanism for the detrimental effect of GH therapy in this context is unclear but possibly related to GH's immunomodulatory effects [22]. However, a critical distinction in our case was that the patient's critically ill condition was a direct consequence of HPS. Therefore, we reasoned that GH could have a therapeutic potential to directly reverse or improve his illness. This approach was considered more favorable than the alternative of proceeding with a high-risk LT, which would be the next step for his very severe HPS. Subcutaneous rhGH was started at 1.0 mg daily, a higher dose than the typical adult starting dose of 0.1 to 0.3 mg/day, because of concern for poor hepatic protein synthetic function in the patient's case. However, this dose is in line with previous rhGH doses in other cases published for panhypopituitarism-related liver disease [2-4] and lower than the 5 to 8 mg daily dose associated with higher mortality in intensive care unit patients treated with rhGH [22].

To our knowledge, this is the first case demonstrating the sustained therapeutic effect of GH in HPS secondary to panhypopituitarism-related MASLD despite extensive liver fibrosis. Beyond adding to the limited literature on successful management of HPS without LT, this observation offers a potential therapeutic target for MASLD-related HPS even in more advanced cases of liver disease. As MASLD is among the leading cause of cirrhosis and LT referral, this novel finding warrants further exploration, with the potential for dual benefit in conserving scarce donor organs and averting transplant-related complications [23].

## Learning Points

- GH is crucial in regulating lipid metabolism in hepatocytes and stimulating IGF-1. Disruption in GH and IGF-1 signaling pathways can lead to development of hepatic steatosis and metabolic dysfunction-associated steatotic liver disease (MASLD).
- Panhypopituitarism with GH deficiency has been associated with hepatopulmonary syndrome (HPS) due to development of MASLD.
- We present a unique case of panhypopituitarism-related MASLD with extensive liver fibrosis complicated by HPS that was successfully treated with GH replacement. This negated the need for liver transplantation, the only established current treatment for HPS.
- We hypothesize that the anti-inflammatory property of GH reversed residual inflammation driving the patient's fibrosis and led to an improvement in HPS.

## Data Availability

Data sharing is not applicable to this article as no datasets were generated or analyzed during the current study.
